# Supplementary material for: A Likelihood Approach for Real-Time Calibration of Stochastic Compartmental Epidemic Models
Source: PLoS Comput Biol. 2017 Jan 17;13(1):e1005257. doi: 10.1371/journal.pcbi.1005257 (PMC5240920; doi:10.1371/journal.pcbi.1005257)
Supplement: S1 File — (TAR.GZ) [file pcbi.1005257.s014.tar.gz › HSPH_Online-SI-Revision/Additions/IPoi10/n100-extreme-SEITR/n100-extreme-SEITR_BENCH2_table-4.pdf]

1	newly 3 weeks spec	13598.	8655.56	{4754., 10186.}	36.3468	0.240685	newly 3 weeks cumul	31455.	23885.3	{9100., 29530.}	24.0651	0.181758	newly 5 weeks spec	12085.	7913.36	{5454., 10122.}	34.5191	0.208674	newly 5 weeks cumul	57301.	40325.2	{24905., 46419.}	29.6257	0.18765	newly 55 weeks cumul	84027.	75685.8	{60631., 89290.}	12.2018	0.0591179
2	newly 3 weeks spec	17235.	3324.27	{326., 7330.}	80.7121	0.960198	newly 3 weeks cumul	29177.	5686.57	{502., 12965.}	80.5101	0.974308	newly 5 weeks spec	17453.	8534.94	{1860., 13768.}	51.0976	0.426581	newly 5 weeks cumul	67910.	20047.5	{3191., 37906.}	70.4793	0.694595	newly 62 weeks cumul	97717.	91944.8	{91185., 92491.}	5.90708	0.0264516
3	newly 3 weeks spec	14424.	7804.88	{1193., 14001.}	47.0252	0.456415	newly 3 weeks cumul	24222.	12864.4	{1644., 24715.}	49.9661	0.492302	newly 5 weeks spec	18266.	14559.1	{8187., 17396.}	20.2942	0.1567	newly 5 weeks cumul	61841.	39742.2	{13232., 57784.}	35.8391	0.291209	newly 64 weeks cumul	97783.	92858.8	{92061., 93492.}	5.03587	0.0224509
4	newly 3 weeks spec	13822.	11755.5	{5061., 15673.}	23.7236	0.155699	newly 3 weeks cumul	25848.	24387.9	{7948., 37875.}	37.1327	0.21636	newly 5 weeks spec	16022.	13198.2	{9103., 15657.}	17.6244	0.0913912	newly 5 weeks cumul	59339.	51243.8	{32104., 62857.}	16.6772	0.0967985	newly 62 weeks cumul	94062.	87456.9	{83948., 90438.}	7.02203	0.0319005
5	newly 3 weeks spec	22578.	6326.14	{1880., 12549.}	71.9809	0.650337	newly 3 weeks cumul	36477.	10208.4	{2696., 21361.}	72.0142	0.667858	newly 5 weeks spec	16920.	14195.8	{10376., 16312.}	16.1006	0.0869062	newly 5 weeks cumul	77798.	35348.	{17862., 52578.}	54.5644	0.378144	newly 64 weeks cumul	97923.	78432.5	{75469., 91150.}	19.9039	0.097247
6	newly 3 weeks spec	20143.	6114.	{1785., 10826.}	69.647	0.631344	newly 3 weeks cumul	34123.	10045.7	{2608., 18613.}	70.5604	0.659224	newly 5 weeks spec	17405.	14107.4	{9311., 16282.}	18.9461	0.130918	newly 5 weeks cumul	74866.	34739.2	{16382., 49819.}	53.5981	0.395304	newly 63 weeks cumul	93450.	89071.9	{86243., 93735.}	4.80923	0.0216453
7	newly 3 weeks spec	12108.	8403.99	{4836., 10190.}	30.5915	0.185252	newly 3 weeks cumul	28975.	22226.5	{9391., 29524.}	24.3002	0.166882	newly 5 weeks spec	11332.	8348.5	{5947., 10191.}	26.3281	0.142149	newly 5 weeks cumul	52923.	39216.5	{25254., 46196.}	25.8989	0.148267	newly 55 weeks cumul	88800.	78160.1	{62521., 89192.}	12.2558	0.0600866
8	newly 3 weeks spec	16980.	6208.79	{919., 13112.}	63.4347	0.647109	newly 3 weeks cumul	32632.	11012.6	{1390., 25977.}	66.252	0.692692	newly 5 weeks spec	14766.	12138.9	{5391., 15116.}	18.7361	0.199181	newly 5 weeks cumul	65812.	32733.7	{9110., 54748.}	50.2618	0.438651	newly 62 weeks cumul	97109.	90757.8	{90344., 93000.}	6.54032	0.0924784
9	newly 3 weeks spec	23304.	2566.22	{289., 5606.}	88.9881	1.16384	newly 3 weeks cumul	35304.	3956.65	{427., 8790.}	88.7926	1.16029	newly 5 weeks spec	17411.	9525.69	{2329., 15227.}	45.2892	0.372502	newly 5 weeks cumul	78660.	19016.8	{3581., 34394.}	75.8241	0.757003	newly 64 weeks cumul	98833.	90965.8	{90262., 92492.}	7.96006	0.0360872
10	newly 3 weeks spec	12030.	11303.1	{7911., 13343.}	15.6649	0.115408	newly 3 weeks cumul	25011.	28882.	{14544., 37710.}	37.4316	0.201381	newly 5 weeks spec	13614.	9984.87	{6589., 12886.}	26.6606	0.150925	newly 5 weeks cumul	52825.	50033.5	{39301., 57042.}	12.5987	0.0830997	newly 60 weeks cumul	95503.	84307.3	{73823., 92429.}	11.7229	0.0561257
11	newly 3 weeks spec	13896.	6150.	{1436., 10214.}	55.7427	0.481431	newly 3 weeks cumul	26326.	12784.7	{2439., 23516.}	52.1585	0.467906	newly 5 weeks spec	14675.	9398.87	{4765., 11251.}	35.9532	0.250777	newly 5 weeks cumul	57151.	30277.4	{9921., 45824.}	47.0222	0.367828	newly 59 weeks cumul	97210.	91182.7	{87767., 93549.}	6.20025	0.0279676
12	newly 3 weeks spec	16108.	7710.58	{2270., 12448.}	52.132	0.387872	newly 3 weeks cumul	33166.	15135.	{3672., 26946.}	54.3941	0.432591	newly 5 weeks spec	14159.	11837.8	{8196., 13385.}	16.3938	0.0856266	newly 5 weeks cumul	64453.	37348.9	{16486., 52493.}	42.0526	0.270603	newly 60 weeks cumul	95054.	90900.5	{88190., 92693.}	4.36966	0.0194977
13	newly 3 weeks spec	15592.	8432.95	{2192., 13500.}	45.9149	0.387837	newly 3 weeks cumul	30732.	17103.7	{3561., 30698.}	46.5665	0.417038	newly 5 weeks spec	14704.	11708.7	{8464., 13901.}	20.371	0.13259	newly 5 weeks cumul	62511.	39544.1	{16797., 56151.}	36.7407	0.270871	newly 61 weeks cumul	96828.	89888.2	{86775., 92281.}	7.16717	0.0324691
14	newly 3 weeks spec	10081.	7620.02	{4587., 9349.}	24.4121	0.196157	newly 3 weeks cumul	23444.	21378.1	{9311., 27287.}	24.7302	0.203143	newly 5 weeks spec	11642.	7041.75	{5325., 9085.}	39.5143	0.258651	newly 5 weeks cumul	46832.	35935.9	{23975., 42613.}	23.2664	0.177313	newly 54 weeks cumul	89533.	71991.5	{58298., 85961.}	19.5922	0.0993657
15	newly 3 weeks spec	14236.	7593.58	{4129., 9700.}	46.6593	0.326369	newly 3 weeks cumul	30841.	18868.4	{8152., 27464.}	38.8205	0.286408	newly 5 weeks spec	13230.	8452.24	{6833., 9678.}	36.1131	0.216128	newly 5 weeks cumul	59486.	35638.6	{22161., 44338.}	40.0891	0.263609	newly 55 weeks cumul	91749.	83641.	{75205., 90311.}	8.86775	0.0417229
16	newly 3 weeks spec	11254.	10212.3	{6875., 11879.}	13.0388	0.0758998	newly 3 weeks cumul	24092.	27304.5	{13208., 33939.}	32.9713	0.153184	newly 5 weeks spec	12953.	9074.59	{5978., 11841.}	29.9422	0.167233	newly 5 weeks cumul	50447.	46398.2	{34813., 52001.}	9.7514	0.0542461	newly 58 weeks cumul	87287.	77366.4	{65795., 88172.}	11.8243	0.0576462
17	newly 3 weeks spec	15615.	3052.89	{416., 5854.}	80.449	0.886728	newly 3 weeks cumul	24517.	4625.24	{618., 8967.}	81.1346	0.910845	newly 5 weeks spec	20574.	11185.8	{3291., 16465.}	45.6313	0.341822	newly 5 weeks cumul	68260.	22441.2	{5059., 37141.}	67.1239	0.590597	newly 64 weeks cumul	92364.	91453.7	{90921., 92964.}	1.24076	0.00541772
18	newly 3 weeks spec	19007.	2713.87	{244., 5733.}	85.7217	1.14906	newly 3 weeks cumul	31170.	4458.56	{419., 9581.}	85.696	1.14229	newly 5 weeks spec	17189.	8396.41	{1667., 14049.}	51.1524	0.512133	newly 5 weeks cumul	70235.	18087.7	{2752., 33828.}	74.2468	0.811954	newly 63 weeks cumul	98793.	89329.7	{88815., 93603.}	9.57894	0.110808
19	newly 3 weeks spec	12889.	4342.35	{428., 7958.}	66.3096	0.647125	newly 3 weeks cumul	24953.	8703.75	{762., 17016.}	65.1194	0.651532	newly 5 weeks spec	14542.	7741.34	{1667., 10665.}	46.7657	0.366923	newly 5 weeks cumul	54787.	22585.4	{3247., 37760.}	58.776	0.518193	newly 58 weeks cumul	96606.	92074.1	{89829., 93657.}	4.6911	0.0209345
20	newly 3 weeks spec	19368.	4662.2	{886., 9289.}	75.9283	0.803212	newly 3 weeks cumul	33454.	7446.36	{1313., 15605.}	77.7415	0.852305	newly 5 weeks spec	16864.	12616.3	{6345., 16283.}	25.1881	0.223624	newly 5 weeks cumul	72041.	28744.7	{10159., 46398.}	60.0995	0.531791	newly 63 weeks cumul	97397.	90708.1	{91040., 92657.}	6.86767	0.117268
21	newly 3 weeks spec	19672.	3475.73	{583., 7525.}	82.3316	0.983137	newly 3 weeks cumul	30439.	5375.15	{837., 11911.}	82.3413	0.997423	newly 5 weeks spec	18543.	11131.3	{4491., 16564.}	39.9706	0.31655	newly 5 weeks cumul	73114.	23522.9	{7050., 41394.}	67.8271	0.628901	newly 64 weeks cumul	98246.	91819.2	{90911., 93390.}	6.54158	0.0294086
22	newly 3 weeks spec	14910.	11808.4	{9620., 13725.}	20.802	0.113334	newly 3 weeks cumul	31688.	30858.7	{17698., 38753.}	23.9562	0.126704	newly 5 weeks spec	14003.	10128.	{6630., 13564.}	27.6724	0.155955	newly 5 weeks cumul	61831.	52506.7	{44537., 58161.}	15.0804	0.0770692	newly 60 weeks cumul	92206.	81529.8	{69276., 90465.}	11.5787	0.0557774
23	newly 3 weeks spec	16317.	10965.8	{3373., 15136.}	32.7951	0.256974	newly 3 weeks cumul	34910.	22612.5	{5297., 36366.}	37.8793	0.312685	newly 5 weeks spec	13260.	12694.5	{10145., 15116.}	13.9514	0.077801	newly 5 weeks cumul	65072.	48155.6	{23783., 61360.}	25.9964	0.175074	newly 61 weeks cumul	83725.	89050.3	{84815., 92659.}	6.75753	0.0282496
24	newly 3 weeks spec	16757.	1858.14	{179., 4651.}	88.9112	1.21757	newly 3 weeks cumul	25641.	3029.62	{279., 7720.}	88.1845	1.20049	newly 5 weeks spec	20012.	6343.64	{1133., 12150.}	68.3008	0.660983	newly 5 weeks cumul	69280.	13101.2	{1824., 28381.}	81.0894	0.921429	newly 63 weeks cumul	98228.	92221.3	{91847., 92501.}	6.1151	0.0274062
25	newly 3 weeks spec	12372.	6004.38	{1914., 10344.}	51.468	0.440711	newly 3 weeks cumul	23919.	12073.3	{3280., 22356.}	51.4107	0.454138	newly 5 weeks spec	15457.	9718.22	{6146., 11846.}	37.1274	0.253307	newly 5 weeks cumul	55191.	29964.7	{13051., 45588.}	45.7072	0.352596	newly 59 weeks cumul	91517.	88118.5	{85689., 89980.}	3.71353	0.0165489
26	newly 3 weeks spec	12283.	8487.29	{3849., 11386.}	30.9021	0.207281	newly 3 weeks cumul	28429.	19032.3	{6949., 28220.}	34.9234	0.249557	newly 5 weeks spec	11730.	10355.1	{8327., 11549.}	11.7236	0.0616006	newly 5 weeks cumul	53475.	39309.6	{22737., 49838.}	26.4897	0.161562	newly 57 weeks cumul	80102.	88223.3	{84433., 92351.}	10.5283	0.04326
27	newly 3 weeks spec	12409.	9305.97	{5761., 11563.}	25.0063	0.171506	newly 3 weeks cumul	28757.	26559.3	{13898., 33136.}	21.9016	0.160992	newly 5 weeks spec	11908.	7765.55	{4628., 11221.}	34.7871	0.210442	newly 5 weeks cumul	53686.	43088.9	{30530., 51000.}	19.7391	0.126728	newly 57 weeks cumul	90968.	72382.3	{54386., 86997.}	20.4311	0.10643
28	newly 3 weeks spec	16959.	13036.6	{3719., 18137.}	26.2449	0.199368	newly 3 weeks cumul	28083.	24359.9	{5337., 39704.}	35.0499	0.242265	newly 5 weeks spec	18063.	14594.2	{10215., 17810.}	19.4399	0.103649	newly 5 weeks cumul	68202.	54541.1	{30919., 66749.}	20.0407	0.121379	newly 64 weeks cumul	88415.	86204.	{81396., 91872.}	5.10949	0.0227409
29	newly 3 weeks spec	12914.	11351.1	{6841., 13916.}	17.1645	0.105486	newly 3 weeks cumul	24569.	26184.4	{12091., 37748.}	35.9813	0.177387	newly 5 weeks spec	15388.	11568.4	{9015., 13811.}	24.8222	0.131915	newly 5 weeks cumul	56007.	50002.8	{36577., 58295.}	13.2246	0.0749352	newly 61 weeks cumul	96532.	86738.7	{80359., 91568.}	10.1451	0.0470905
30	newly 3 weeks spec	13125.	2823.56	{526., 5723.}	78.4872	0.822576	newly 3 weeks cumul	20142.	4323.86	{743., 8726.}	78.5331	0.831243	newly 5 weeks spec	19690.	10444.1	{3779., 15559.}	46.9573	0.341587	newly 5 weeks cumul	60177.	20900.1	{6062., 35207.}	65.269	0.553634	newly 64 weeks cumul	98710.	92286.9	{92071., 92575.}	6.50708	0.0292271
31	newly 3 weeks spec	10370.	8698.12	{6951., 10196.}	16.1223	0.11659	newly 3 weeks cumul	26082.	24792.	{14627., 29635.}	16.2852	0.119334	newly 5 weeks spec	9931.	7610.16	{5034., 10166.}	24.1806	0.155603	newly 5 weeks cumul	46868.	40785.8	{33692., 45999.}	12.9773	0.0931196	newly 54 weeks cumul	76589.	72081.9	{54354., 87445.}	14.0759	0.0666349
32	newly 3 weeks spec	12614.	11371.4	{5909., 13978.}	17.2924	0.119419	newly 3 weeks cumul	26998.	26732.6	{10035., 38882.}	32.5764	0.190658	newly 5 weeks spec	13001.	11241.7	{7550., 13676.}	16.6099	0.0952362	newly 5 weeks cumul	54036.	50059.7	{33505., 58592.}	13.7103	0.0870912	newly 60 weeks cumul	93822.	85795.8	{76947., 91601.}	8.55471	0.0398739
33	newly 3 weeks spec	20264.	16846.5	{13177., 19681.}	17.0439	0.097484	newly 3 weeks cumul	37971.	44034.1	{28407., 52626.}	28.6184	0.132838	newly 5 weeks spec	15166.	9975.89	{5292., 15929.}	37.8434	0.231891	newly 5 weeks cumul	73450.	67977.9	{61381., 72839.}	7.45005	0.037825	newly 64 weeks cumul	96763.	83951.5	{72127., 91982.}	13.2401	0.0635682
34	newly 3 weeks spec	15602.	11564.6	{6304., 13930.}	25.8772	0.161998	newly 3 weeks cumul	34185.	27680.3	{10916., 39033.}	27.0183	0.182525	newly 5 weeks spec	13645.	11140.7	{8091., 13980.}	19.1584	0.0998731	newly 5 weeks cumul	63916.	50838.1	{34760., 58973.}	20.461	0.115373	newly 60 weeks cumul	89965.	85491.4	{77741., 91629.}	5.72889	0.0262974
35	newly 3 weeks spec	18683.	3449.73	{506., 8432.}	81.5355	0.966997	newly 3 weeks cumul	29482.	5324.8	{729., 13505.}	81.9388	0.993121	newly 5 weeks spec	19217.	11582.6	{3719., 16527.}	39.7273	0.354582	newly 5 weeks cumul	73185.	24057.8	{5879., 44827.}	67.1274	0.654728	newly 64 weeks cumul	94833.	89788.4	{90719., 91742.}	5.31941	0.117355
36	newly 3 weeks spec	11929.	3537.93	{491., 6654.}	70.3417	0.69633	newly 3 weeks cumul	21930.	6566.28	{834., 12682.}	70.3014	0.70798	newly 5 weeks spec	15024.	7909.44	{2215., 11484.}	47.3546	0.36704	newly 5 weeks cumul	52068.	20111.5	{4102., 34110.}	61.3745	0.535174	newly 60 weeks cumul	97587.	89338.5	{88270., 90293.}	8.45248	0.0383777
37	newly 3 weeks spec	15965.	2074.17	{73., 3993.}	87.008	1.18744	newly 3 weeks cumul	25084.	3729.07	{122., 7029.}	85.1337	1.13068	newly 5 weeks spec	18951.	5472.4	{335., 9428.}	71.1234	0.744354	newly 5 weeks cumul	65780.	12781.8	{601., 23107.}	80.5689	0.951886	newly 61 weeks cumul	98650.	88815.7	{87733., 89188.}	9.96886	0.0456616
38	newly 3 weeks spec	14686.	9727.13	{6038., 11908.}	33.766	0.209385	newly 3 weeks cumul	31691.	23546.4	{11494., 33703.}	27.9278	0.190732	newly 5 weeks spec	13535.	10142.1	{7743., 11805.}	25.0673	0.131422	newly 5 weeks cumul	60924.	44136.6	{31695., 52058.}	27.5547	0.157741	newly 58 weeks cumul	87770.	82709.7	{74010., 88750.}	6.38534	0.029511
39	newly 3 weeks spec	14352.	9387.73	{2779., 13198.}	34.5894	0.259542	newly 3 weeks cumul	28679.	20262.6	{4589., 33814.}	36.8856	0.28516	newly 5 weeks spec	15042.	11438.8	{9046., 13233.}	23.9543	0.133952	newly 5 weeks cumul	60279.	42800.	{19406., 56394.}	28.9968	0.191218	newly 60 weeks cumul	93593.	90158.5	{85074., 93606.}	3.72691	0.0168179
40	newly 3 weeks spec	14498.	13279.	{11227., 15107.}	10.8407	0.0628186	newly 3 weeks cumul	31327.	35337.	{23000., 42243.}	27.087	0.127309	newly 5 weeks spec	13194.	9958.44	{6581., 13674.}	26.8456	0.148806	newly 5 weeks cumul	59514.	57429.5	{50473., 62261.}	6.95142	0.0365227	newly 61 weeks cumul	94658.	81849.9	{70982., 90660.}	13.5309	0.0653319
41	newly 3 weeks spec	13435.	1179.23	{104., 3027.}	91.2227	1.26148	newly 3 weeks cumul	19619.	1851.42	{165., 4678.}	90.5631	1.23386	newly 5 weeks spec	23349.	5230.22	{760., 10448.}	77.5998	0.782348	newly 5 weeks cumul	66595.	9738.14	{1201., 21230.}	85.3771	0.990772	newly 64 weeks cumul	95882.	92782.7	{92605., 93089.}	3.23237	0.0142911
42	newly 3 weeks spec	10315.	8955.62	{6841., 10248.}	13.3199	0.0778728	newly 3 weeks cumul	25161.	23418.9	{14201., 29319.}	19.5295	0.111637	newly 5 weeks spec	10541.	8715.02	{6521., 10127.}	17.3227	0.0889312	newly 5 weeks cumul	46907.	41299.	{33228., 46284.}	11.9814	0.0660789	newly 55 weeks cumul	87719.	78825.3	{67713., 87986.}	10.4629	0.0501895
43	newly 3 weeks spec	12045.	10948.4	{5673., 13334.}	17.6758	0.116581	newly 3 weeks cumul	24152.	28837.6	{9865., 37379.}	45.3606	0.220126	newly 5 weeks spec	14237.	9518.22	{6711., 13030.}	33.1445	0.188329	newly 5 weeks cumul	52694.	48990.5	{31409., 56553.}	13.7613	0.0815105	newly 60 weeks cumul	95113.	79952.6	{69322., 90402.}	15.9393	0.0782311
44	newly 3 weeks spec	16196.	6800.16	{970., 12415.}	58.0133	0.523675	newly 3 weeks cumul	32797.	13436.9	{1617., 27416.}	59.248	0.564519	newly 5 weeks spec	14170.	10718.5	{4616., 13161.}	24.3581	0.168861	newly 5 weeks cumul	64055.	33355.9	{8473., 52335.}	47.9262	0.374561	newly 60 weeks cumul	95957.	89900.1	{86408., 91747.}	6.31211	0.0284359
45	newly 3 weeks spec	16795.	6182.29	{1483., 11278.}	63.1897	0.576203	newly 3 weeks cumul	31747.	10629.6	{2276., 20856.}	66.5178	0.636069	newly 5 weeks spec	14996.	12551.4	{7589., 15196.}	16.88	0.126455	newly 5 weeks cumul	65154.	33030.4	{13619., 50064.}	49.3041	0.375647	newly 62 weeks cumul	96985.	92286.2	{91227., 92990.}	4.8449	0.0215786
46	newly 3 weeks spec	15111.	12908.	{4680., 17227.}	24.2246	0.161744	newly 3 weeks cumul	25292.	25545.2	{6876., 40314.}	40.3438	0.224438	newly 5 weeks spec	17991.	14251.5	{11001., 17096.}	20.7852	0.107458	newly 5 weeks cumul	63693.	54850.3	{33821., 67127.}	16.1789	0.0938213	newly 64 weeks cumul	97957.	91147.4	{88398., 93306.}	6.9516	0.0314133
47	newly 3 weeks spec	13356.	8681.22	{2082., 12188.}	35.0013	0.264835	newly 3 weeks cumul	27095.	20337.5	{3408., 34482.}	37.7199	0.288922	newly 5 weeks spec	14547.	9996.81	{6499., 12367.}	31.2792	0.178774	newly 5 weeks cumul	57180.	40128.3	{13786., 53218.}	29.8212	0.201483	newly 59 weeks cumul	92154.	84175.4	{74965., 89607.}	8.65785	0.0403425
48	newly 3 weeks spec	14508.	8701.69	{2777., 13501.}	40.0545	0.282146	newly 3 weeks cumul	25267.	16529.4	{4368., 28856.}	40.844	0.291025	newly 5 weeks spec	17877.	13168.	{10381., 14619.}	26.341	0.138306	newly 5 weeks cumul	62499.	41487.9	{20438., 56041.}	33.6182	0.204201	newly 62 weeks cumul	95356.	91739.9	{89501., 93307.}	3.79224	0.0168599
49	newly 3 weeks spec	15110.	12186.8	{9296., 14371.}	19.3462	0.0995401	newly 3 weeks cumul	34829.	36745.3	{29876., 40680.}	13.4818	0.0607512	newly 5 weeks spec	12499.	8069.71	{4978., 12170.}	36.6742	0.219663	newly 5 weeks cumul	62570.	55148.5	{47688., 60353.}	11.8612	0.0571576	newly 60 weeks cumul	88106.	74358.8	{59559., 88891.}	16.1306	0.0811769
50	newly 3 weeks spec	13796.	11392.4	{5940., 14609.}	20.5159	0.132918	newly 3 weeks cumul	28249.	24776.7	{9841., 37346.}	31.348	0.191202	newly 5 weeks spec	14245.	12393.3	{9889., 14523.}	13.6614	0.0684713	newly 5 weeks cumul	58206.	49990.3	{34317., 60380.}	15.8679	0.0901508	newly 61 weeks cumul	94140.	89433.9	{84644., 92957.}	4.99903	0.0226279
